# Pahari POS-tagged corpus: A large-scale linguistic resource for NLP applications

**DOI:** 10.1016/j.dib.2026.112515

**Published:** 2026-02-03

**Authors:** Nadia Mushtaq Gardazi, Muhammad Kamran Malik, Ali Daud

**Affiliations:** a Department of Computer Science, Faculty of Computing and Information Technology, University of the Punjab, Lahore, Pakistan; b Department of Data Science, Faculty of Computing and Information Technology, University of the Punjab, Lahore, Pakistan; c Faculty of Resilience, Rabdan Academy, Abu Dhabi, United Arab Emirates

**Keywords:** Low-resource language, Pahari language, Part-of-speech tagging, POS tag set, Natural language processing (NLP), Corpus annotation, South Asian language

## Abstract

This paper presents the development of a Part-of-Speech (POS) tagged dataset for Pahari, an under-resourced Indo-Aryan language spoken in Azad Jammu and Kashmir, Pakistan, as well as parts of India and Nepal. The lack of linguistic resources for Pahari has hindered the advancement of Natural Language Processing (NLP) tools, limiting its computational analysis. This study addresses this gap by creating a POS-tagged dataset, defining a tag set tailored to Pahari, and establishing annotation guidelines. The Pahari POS tag set was designed by leveraging existing tag sets from Urdu, Hindi, Punjabi, and other Indo-Aryan languages, ensuring linguistic compatibility. A corpus of 200,000 tokens was collected and manually annotated, achieving an inter-annotator agreement of 92.3 % (Cohen’s Kappa). This paper explores the key challenges faced during data collection, preprocessing, and annotation, and details the methodologies employed to address them. The resulting dataset represents the first structured linguistic resource developed for Natural Language Processing (NLP) in the Pahari language. It lays a critical foundation for future research in areas such as morphosyntactic analysis, Named Entity Recognition (NER), and the development of machine learning-based NLP applications.

## 1 Introduction

Natural Language Processing (NLP) has seen remarkable progress in high-resource languages such as English, Chinese, and French, largely due to the availability of extensive annotated corpora and sophisticated linguistic tools. However, low-resource languages, such as Pahari, remain largely unexplored due to the lack of annotated datasets, linguistic resources, and standardized tools. Pahari, spoken by over 5-6 million people across Pakistan, India, Nepal, and diaspora communities in the United Kingdom, is one such language that has received minimal attention in the NLP community despite its wide usage and linguistic richness. The Pahari language, a member of the Indo-Aryan language family, is spoken predominantly in the hilly regions of Pakistan and India. In his seminal work ‘Urdu aur Pahari ke Taqabli Jaeza’, Karnai [1] identifies four main dialects of Western Pahari: Muzaffarabad, Poonch, Kotli, and Mirpuri. The Muzaffarabadi dialect is spoken in Muzaffarabad, with slight variations in Anantnag, Baramula, and Srinagar, influenced by Hindko and Kashmiri. The Mirpuri dialect, influenced by Gojri and Punjabi, is prevalent in Mirpur, Bhimber, and Kotli districts, as well as Jammu and Rajouri. The Kotli dialect, spoken in Kotli, Nikyal, Khoiratta, and Nibaa Valley, shows influences from Gojri and Potohari. Finally, the Poonch dialect, referred to as the central dialect, is spoken in Poonch, Bagh, Sudhanhoti, and Haveli districts. Despite the significant number of speakers, obtaining plain text in Pahari for linguistic analysis has been challenging due to the lack of effective resources. As a result, our dataset comprises a combination of texts from different dialects, which may impact the accuracy of our results. In future research, we aim to perform tasks separately on individual dialects once sufficient plain text is available.

This paper focuses on the development of a Part-of-Speech (POS) tagged dataset for Pahari, a critical first step in building foundational NLP tools for this language. POS tagging, the process of assigning grammatical categories (e.g., noun, verb, adjective) to words in a text, is a fundamental task in NLP. Part-of-Speech (POS) tagging serves as a foundational step for more advanced NLP tasks such as Named Entity Recognition (NER), machine translation, and text-to-speech synthesis. However, the development of accurate POS taggers relies heavily on the availability of well-defined tag sets and annotated corporaresources that are particularly scarce for low-resource languages. In South Asia, languages like Urdu, Hindi, Punjabi, Sindhi, and Pashto, spoken widely across Pakistan and India have witnessed some progress in POS tagging. Yet, they continue to face major hurdles due to their morphological complexity, inconsistent standardization, and limited annotated datasets. Even more marginalized languages, such as Pahari, present further challenges, including the absence of capitalization norms, substantial dialectal variation, and a near-complete lack of prior computational work. This study seeks to bridge this gap by developing a POS-tagged dataset for Pahari, laying the groundwork for future NLP research in this under-resourced linguistic domain. The primary contributions of this paper are:

• Development of a POS-tagged dataset for the Pahari language, addressing a critical gap in linguistic resources for this under-resourced language.
• Design of a custom tag set tailored to the morphological and syntactic features specific to Pahari, enabling more accurate linguistic annotation and analysis.
• Annotation guidelines and the methodology used to ensure consistency and accuracy in tagging.
• A discussion of the challenges faced during data collection, cleaning, and annotation, along with the solutions implemented.

**Fig. 1:**
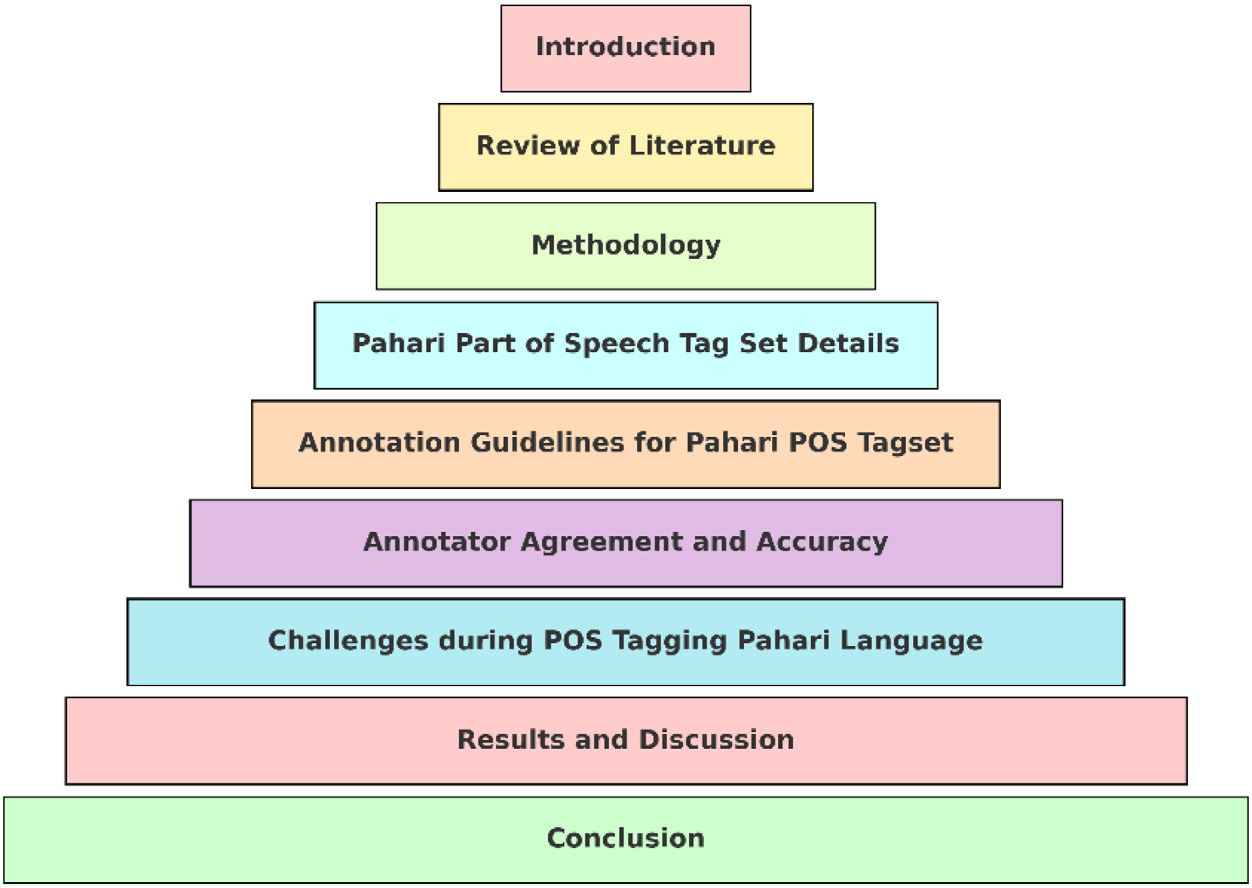
Organization of Pahari Part of Speech tagging.

This study includes the introduction of the problem, a review of past research, the design of a customized Pahari POS tag set, annotation guidelines, and annotator agreement results. It discusses key challenges during tagging, presents the results and linguistic insights, and concludes with the significance of the work for future NLP and language preservation efforts. The details of section is shown in the Fig. 1 below.

## 2 Review of Literature

Part-of-Speech (POS) tagging is a fundamental task in Natural Language Processing (NLP) that involves assigning grammatical categories (e.g., noun, verb, adjective) to words in a text. While POS tagging is well-studied for high-resource languages like English, Chinese, and French, low-resource languages face significant challenges due to the lack of annotated corpora, linguistic tools, and standardized writing systems. This section reviews existing work on POS tagging and dataset creation for low-resource languages, with a focus on Indo-Aryan languages and other morphologically rich languages.

### 2.1 Annotated corpora for Indo-Aryan languages

A variety of annotated corpora have been developed for Indo-Aryan languages such as Urdu, Hindi, Punjabi, Sindhi, Pashto, Bengali, Nepali, and others. Comparing POS tagging resources and methodologies across these languages reveals both common strategies and language-specific adaptations.

• **Hindi and Urdu:** These languages have the largest annotated corpora, with millions of tokens, thanks to early initiatives like the EMILLE corpus, the Hindi Dependency Treebank (HDTB), and the Indian Languages Corpora Initiative (ILCI) [2,3]. The Bharatiya Indian Standard (BIS) tagset, consisting of approximately 35 POS tags, is widely adopted for Hindi. POS tagsets developed for Urdu range from highly detailed systems with around 350 tags [3] to simplified versions using just 42 tags [4].
• **Bengali, Punjabi, and Nepali**: These languages have moderately sized annotated corpora. For example, the Bengali POS Tagged Corpus includes around 45,000 tokens [5], and the Punjabi Dependency Treebank contains approximately 50,000 tokens [6]. The Universal Dependencies (UD) initiative has also produced treebanks for these languages, employing a streamlined set of 17 universal POS tags [7].
• **Sindhi and Pashto**: These languages have smaller POS-tagged resources. The Sindhi corpus contains around 30,000 tokens [8], while the Pashto corpus comprises roughly 20,000 tokens [9]. Both face substantial limitations due to their low-resource status and lack of standardized annotation tools.

### 2.2 Proposed methodology

**Fig. 2:**
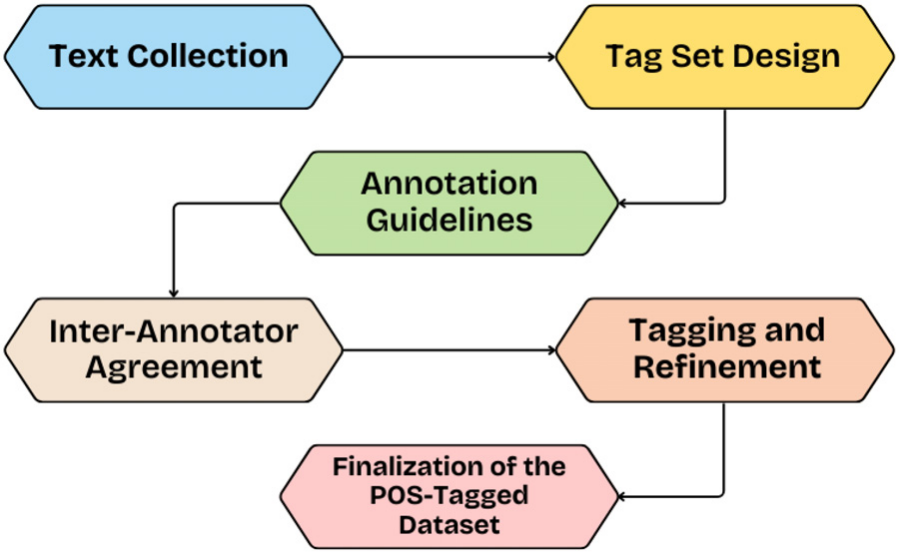
Methodology of Pahari POS Tag set.

The construction of the POS-tagged dataset for the Pahari language was carried out through a systematic, six-phase process as shown in the Fig. 2:

1. **Text Collection**: Raw textual data was gathered from diverse sources in Pahari, including folk tales, interviews, casual dialogues, and informal conversations. The distribution of data sources included in the corpus is presented in Table 1.

**Table 1:** Distribution of Data Sources in the Pahari POS Corpus.

Source Type	Description	Percentage of Corpus
News Articles	Online news reports, magazines, newspapers	30 %
Folk Stories	Oral stories, folk tales	25 %
Other Written Stories	Short stories, general narratives, written literature	25 %
Conversational Speech & Interviews	Natural dialogues, spoken interviews, spontaneous speech	20 %
  This variety ensured linguistic richness and contextual diversity.
2. **Tagset Design:** A customized POS tagset was developed by drawing inspiration from established Urdu tagsetssuch as those by Sajjad [4], Muaz (2009), and Ahmed et al. (2015). Additionally, linguistic features specific to Pahari were incorporated using insights from Karnai [1], ensuring both computational applicability and linguistic relevance.
3. **Annotation Guidelines and Initial Annotation:** Comprehensive guidelines were prepared to guide annotators and ensure consistency. A pilot annotation was then performed on a subset of the data to test the clarity and adequacy of the tag definitions.
4. **Validation through Inter-Annotator Agreement:** To assess the reliability of annotations, inter-annotator agreement metrics were calculated. Disagreements were systematically analyzed, prompting revisions to the guidelines to reduce ambiguity and enhance consistency.
5. **Tagging and Refinement:** Once validated, the dataset underwent large-scale annotation. Continuous feedback loops were established, allowing for regular correction of errors, handling of edge cases, and iterative refinement of the tagset and annotation practices.
6. **Finalization of the POS-Tagged Dataset:** Through collaborative review and iterative adjustments, the final version of the POS-tagged Pahari dataset was produced. It is both linguistically robust and suitable for computational applications in NLP research.

## 3 Challenges in POS Tagging for Indo-Aryan Languages

POS tagging for Indo-Aryan languages poses significant challenges due to script variation, dialectal diversity, and complex morphological structures. These challenges become even more pronounced in low-resource languages, where annotated corpora and NLP tools are scarce [3,8,9]. In this study, we adapted existing Urdu POS tagsets particularly those developed by Sajjad [4], Muaz (2009), and Ahmed et al. (2015), to create a linguistically compatible and computationally viable tagset for Pahari. This adaptation was facilitated by close linguistic and historical ties between Urdu and Pahari. Additionally, substantial guidance was drawn from Karnai’s (2007) work *Pahari aur Urdu: Ik Taqabali Jaiza*, which offers detailed comparative analysis of both languages. His classification of grammatical categories played a pivotal role in informing structure and scope of the proposed tagset.

## 4 Pahari Part of Speech tagset Details

**Fig. 3:**
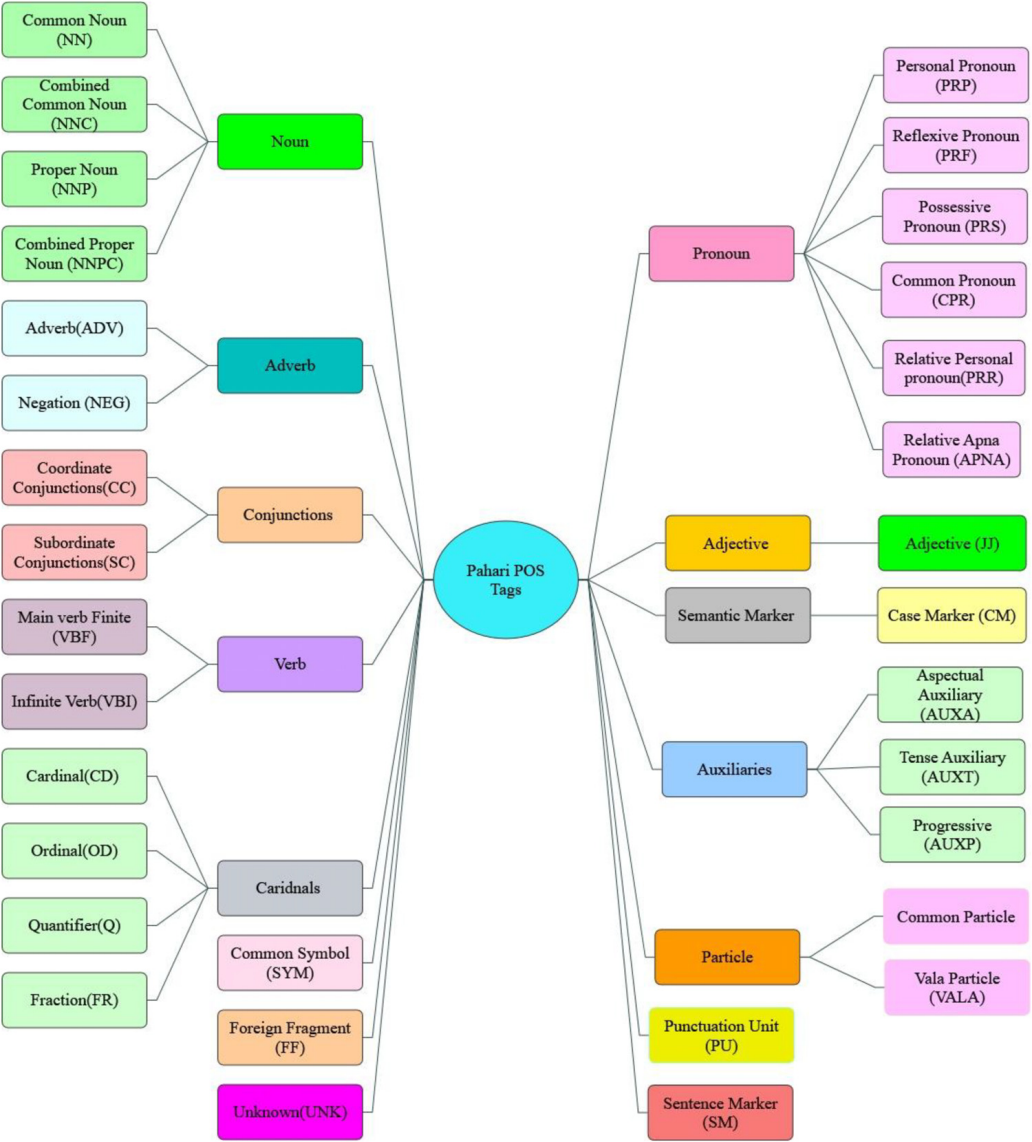
Final Pahari Tag set.

Developing a POS tagset is essential for linguistic analysis, syntactic parsing, and various downstream NLP applications. For Pahari, a comprehensive POS tagset was designed to support accurate annotation and meaningful linguistic representation as presented in the Fig. 3. Its foundational structure was informed by Karnai’s (2007) work *Pahari aur Urdu: Ik Taqabali Jaiza*, which offers a comparative analysis of Pahari and Urdu grammar. To ensure both linguistic coherence and computational feasibility, the Pahari tagset was aligned with previously developed Urdu POS tagsets, including those by Muaz, Ali, and Hussain (2009), Sajjad and Schmid (2009), and Ahmed et al. (2015). The design also incorporated principles from the CLE Urdu POS tagset. Furthermore, the framework was influenced by Penn Treebank guidelines [10], ensuring alignment with widely accepted linguistic annotation standards.

The Pahari POS tagset is a structured, hierarchical system covering major morpho-syntactic categories. It balances linguistic precision with computational efficiency, supporting both manual and automatic tagging. Categories are defined to reflect Pahari-specific features while ensuring compatibility with Urdu and related South Asian languages.

## 5 Annotation Guidelinesfor Paharipos Tagset

### 5.1 Noun (common noun, proper noun)

Nouns in Pahari, as in other Indo-Aryan languages, represent entities such as people, places, objects, animals, and abstract concepts. While Karnai [1] identified five noun types common, abstract, collective, material, and uncountable for linguistic categorization, we adopt a simplified four-category classification in the Pahari POS tagset to align with computational frameworks. This decision was informed by a comparative analysis of prominent Urdu POS tagsets: Sajjad [4], Muaz (2009), and Ahmed et al. (2015).

For common nouns, all three Urdu tagsets consistently use the label *NN*. For proper nouns, Muaz and Ahmed et al. employ *NNP*, whereas Sajjad uses *PN*. We primarily adopted the Muaz et al. (2009) conventions due to their consistency and utility in Urdu NLP applications. To enhance handling of multiword noun expressions a common feature in Pahari we introduced two additional tags: *NNC* (Common Noun Combined) and *NNPC* (Proper Noun Combined), facilitating accurate annotation of compound noun structures. Examples are provided in Table 2.

**Table 2:** Examples of Nouns in the Pahari POS Tagset.

### 5.2 Pronoun

#### 5.2.1 Personal pronoun (PRP)

In Pahari, personal pronouns reflect distinctions in number and formality (e.g., "" vs. ""). Based on Karnai [1], various pronoun types exist in the language, but for computational simplicity, we consolidated personal, demonstrative, and exclamatory pronouns under a single ta PRP. Among Urdu POS tag sets, Ahmed et al. (2015) employed a more structured approach by using the PRP tag, which we adopted for consistency and interoperability with standard NLP frameworks.

#### 5.2.2 Possessive pronoun(PRS)

Possessive pronouns in Pahari indicate ownership and agree with the nouns they modify. Sajjad [4] used *G*, Muaz (2009) used *PRP$*, and Ahmed et al. (2015) used *PRS*. As *PRS* is widely adopted in modern NLP, we selected it for the Pahari tag set. Examples are provided in Table 3.

**Table 3:** Examples of Pronouns in the Pahari POS Tagset.

#### 5.2.3 Reflexive pronoun(PRF)

Reflexive pronouns refer back to the subject of a sentence. Karnai [1] categorized them under retroflexive pronouns in Pahari, but in NLP, they are standardized as reflexive pronouns (*PRF*). Sajjad [4] used *RP*, Muaz (2009) introduced *PRRF*, while Ahmed et al. (2015) adopted *PRF*. To maintain consistency with modern tagging practices, we used *PRF* for reflexive pronouns in the Pahari tag set. Examples are provided in Table 3.

#### 5.2.4 Reflexive Apnapronoun(APNA)

The Reflexive *Apna* pronoun indicates possession in a self-referential manner. Although Karnai [1] treated it as a separate category, we followed Urdu NLP conventions by tagging it as *APNA*. Sajjad [4] grouped it under general possessives (*GR*), while Muaz (2009) used *PRRFP$*, merging it with relative pronouns, creating ambiguity. Ahmed et al. (2015) correctly treated *APNA* as a distinct class, which we adopted for the Pahari tagset to ensure clarity and linguistic precision. Table 3 presents examples of *APNA* in Pahari, Urdu, and English.

#### 5.2.5 Relative personalpronoun(PRR)

Relative personal pronouns refer to a previously mentioned noun and link clauses to add information. Common in complex Pahari and Urdu sentences, they are tagged as *PRR* following Ahmed et al. (2015), whose classification aligns with standard NLP practices. Table 3 presents examples of *PRR* in Pahari, Urdu, and English.

#### 5.2.6 Common pronoun(CPR)

Common pronouns refer to indefinite or unspecified entities (e.g., ). These forms are essential for expressing generalization or uncertainty in Pahari. All three Urdu POS tagsets. Sajjad [4], Muaz (2009), and Ahmed et al. (2015) consistently use *CPR* for this category. To maintain compatibility and standardization, we adopted *CPR* for Pahari as well. Table 3 presents examples of *CPR* in Pahari, Urdu, and English.

### 5.3 Adjectives

Adjectives describe or qualify nouns and pronouns. In Pahari, they agree in number and definiteness with the noun but show limited gender inflection compared to Urdu. We followed Muaz (2009) and Ahmed et al. (2015) in adopting *JJ* as the standard tag, covering descriptive, degree-based, temporal, and spatial adjectives. Table 4 presents examples of *JJ* in Pahari, Urdu, and English.

**Table 4:** Examples of Adjective (JJ) in the Pahari POS Tagset.

### 5.4 Adverbs

#### 5.4.1 Adverb

Adverbs modify verbs, adjectives, or entire phrases. In Pahari, they appear by manner (), place (), time (), and degree (). We adopted *ADV* as the unified tag, aligning with Sajjad [4] and refining beyond Muaz’s (2009) *RB*. Table 5 shows examples in Pahari, Urdu, and English.

**Table 5:** Examples of Adverb (ADV) and Negation (NEG) in the Pahari POS Tagset.

#### 5.4.2 Negation

Negation plays a critical role in modifying sentence meanings by negating a statement. In the Pahari POS Tag set, negation words such as "" are categorized under the NEG (Negation) tag. Both Sajjad [4] and Ahmed et al. (2015) included NEG as a distinct tag for negation, ensuring clarity and consistency. Muaz (2009) did not define it separately, but for the Final Pahari Tag set, we adopted the NEG tag to maintain linguistic precision and computational ease. Examples are presented in Table 5.

### 5.5 Conjunctions (CC, SC)

A conjunction is a word that connects words, phrases, clauses, or sentences. In Pahari, as in Urdu, conjunctions are divided into two types (Coordinating Conjunctions (CC) and Subordinating Conjunctions (SC)). Sajjad [4] Tagset categorized conjunctions into CC and SC but provided only a basic structure without deeper classification. Muaz (2009) followed the same classification, ensuring compatibility but without any syntactic refinements. Ahmed et al. (2015) maintained CC and SC, aligning with standard Urdu linguistic structures, making it more stable for NLP applications.

### 5.6 Verbs

A verb is a word that represents an action, occurrence, or state of being. In Pahari and Urdu, verbs are categorized into main verbs and auxiliaries. Examples of All verb types are present in Table 6.

**Table 6:** Examples of Verb Tags in the Pahari POS Tag set.

#### 5.6.1 Finite verbs (VBF)

Finite verbs are marked for tense, number, and subject. While earlier Urdu tagsets (Sajjad, Muaz) used a general *VB* tag, Ahmed et al. (2015) introduced *VBF* to distinguish finite verbs. We adopt *VBF* for syntactic clarity.

#### 5.6.2 Infinitive verbs (VBI)

Infinitives (e.g., ) lack tense or agreement. All three Urdu tagsets used *VBI*. We adopted the same for Pahari for consistency and clear verb type separation.

#### 5.6.3 **Auxiliary verbs (**AUXA**)**

Auxiliaries expressing aspect (e.g., ) are tagged *AUXA*. Following Muaz (2009) and Ahmed et al. (2015), we use *AUXA* to annotate aspectual functions precisely.

#### 5.6.4 **Tense auxiliaries (**AUXT**)**

Auxiliaries indicating tense (e.g., ) are tagged as *AUXT*. We follow Muaz and Ahmed et al., aligning with international NLP frameworks.

#### 5.6.5 Progressive auxiliaries(AUXP)

To mark ongoing actions (e.g., ), we use *AUXP*. This tag, introduced by Muaz (2009) and retained by Ahmed et al. (2015), improves aspectual annotation.

### 5.7 Numbers

#### 5.7.1 Cardinal numbers (CD)

Cardinal numbers represent countable quantities and answer the question "How many?" in a sentence. These numbers do not indicate order but rather the total count of objects. Table 7 presents Cardinal Numbers (CD) in Pahari, Urdu, and English**.**

**Table 7:** Example of Cardinal Numbers.

Sajjad [4], Muaz (2009), and Ahmed et al. (2015) all used CD, making it the standard classification for cardinal numbers. Since all previous tag sets consistently used CD, we adopted it for Pahari to maintain linguistic clarity and computational efficiency.

#### 5.7.2 Ordinal numbers(OD)

Ordinal numbers indicate position or rank rather than count. They answer the question "Which one in order?" and are used to describe sequence. Table 8 presents Ordinal Numbers (OD) in Pahari, Urdu, and English**.**

**Table 8:** Examples od Ordinal Numbers.

All previous Urdu POS tag sets consistently used OD for ordinal numbers, making it the preferred classification. To ensure consistency with Urdu and improve computational tagging, we adopted OD for the Pahari POS tag set.

#### 5.7.3 Quantifiers (Q)

Quantifiers specify an indefinite quantity of something rather than an exact number. They describe "How much?" or "How many?" without being precise. Table 9 presents Quantifiers (Q) in Pahari, Urdu, and English**.**

**Table 9:** Examples of Quantifiers.

All previous Urdu POS tag sets classified quantifiers as Q**,** making it a linguistically accurate choice**.** To maintain compatibility with Urdu POS structures and NLP applications, we adopted Q for the Pahari POS tag set.

#### 5.7.4 Fractional numbers(FR)

Fractional numbers express a part of a whole and typically include terms like half, one-third, three-quarters, etc. Table 10 represents Fractional Numbers (FR) in Pahari, Urdu, and English**.**

**Table 10:** Examples of Fractional Numbers.

Sajjad [4], Muaz (2009), and Ahmed et al. (2015) consistently used FR for fractional numbers. To ensure NLP accuracy and computational efficiency, we adopted FR for the Pahari POS tag set.

### 5.8 Particle

#### 5.8.1 Common particle(PRT)

Common particles in Pahari include functional elements like  and emphatic expressions like , which don't change form and add emphasis or connectivity to a sentence. Table 11 represents **Common Particle (PRT)** in Pahari, Urdu, and English**.**

**Table 11:** Example of Common Particle.

We adopted PRT from Sajjad [4] and Ahmed et al. (2015) for Pahari to retain consistency in tagging functional grammatical elements.

#### 5.8.2 Vala particle(VALA)

VALA-type particles like  are attributive and used for emphasis or specification, often functioning adjectivally. Table 12 shows Vala Particle (VALA) in Pahari, Urdu, and English**.**

**Table 12:** Example of VALA particle.

We retained VALA as a separate tag, aligning with the structure of Ahmed et al. (2015) and ensuring clarity for these contextually rich particles.

### 5.9 Interjection (INT)

Interjections are spontaneous expressions of emotion, reaction, or exclamation that stand independently in a sentence. They often convey feelings like surprise, admiration, sorrow, or urgency. In Pahari (as well as in Urdu), these include religious, emotional, or conversational outbursts such as: . Table 13 presents example of interjections.

**Table 13:** Example of Interjections.

Sajjad [4] used INT, while both Muaz (2009) and Ahmed et al. (2015) used INJ. For simplicity and abbreviation consistency across the tag set, INT was adopted in the Final Pahari Tag set.

### 5.10 Semantic marker (CM)

Semantic markers, often known as postpositions or case markers, are grammatical tools in the Pahari language that show relationships like possession, location, direction, comparison, and cause. They usually follow nouns or pronouns and function similarly to Urdu’s postpositions like . Table 14 represents Vala Particle (VALA) in Pahari, Urdu, and English**.**

**Table 14:** Example of Semantic Marker.

### 5.11 Symbol (SYM)

Common symbols refer to characters such as **+, %, $,**  that occur within text but are not treated as words. They are important in numeric, financial, and temporal expressions, especially in formal and academic writing. Table 15 shows the examples of Symbol (SYM) in Pahari, Urdu, and English**.**

**Table 15:** Examples of Symbol in Pahari.

All three Urdu POS tag sets, Sajjad [4], Muaz (2009), and Ahmed et al. (2015) common symbols are classified under the tag SYM.

### 5.12 Punctuation unit (PU)

The Punctuation Unit (PU) tag is used to annotate punctuation marks such as  ! () / etc. These symbols do not hold lexical meaning but play a crucial role in sentence structure, intonation, and discourse clarity. They help segment text into meaningful units and guide syntactic interpretation. Table 16 shows Punctation Unit (PU) in Pahari, Urdu, and English**.**

**Table 16:** Example of Punctuation Unit.

All three Urdu tag sets consistently used PU to mark punctuation. This consistency made it straightforward to adopt the PU tag for the Final Pahari Tag set as well.

### 5.13 Foreign fragment (FF)

The Foreign Fragment (FF) tag is used to annotate words, phrases, or full expressions written in a foreign language (typically Arabic, English, or Persian) that appear within a native Pahari or Urdu sentence. Table 17 represents Foreign Fragment (FF) in Pahari, Urdu, and English**.**

**Table 17:** Foreign Fragment in Pahari Language.

Both Sajjad [4] and Ahmed et al. (2015) explicitly included the FF tag to handle foreign language fragments, while Muaz (2009) did not distinguish them separately, possibly grouping them under general or unknown categories. Therefore, the Final Pahari Tag set adopted the FF tag to ensure clarity and consistency, particularly for Arabic and English insertions.

## 6 Annotator Agreementand Accuracy

To ensure the reliability and accuracy of our Part of Speech (POS) tag set for Pahari, we conducted an extensive evaluation involving two native speaker annotators. These annotators meticulously reviewed and tagged a substantial corpus of Pahari text, achieving an impressive agreement rate of 92 %. This high level of concordance underscores the robustness and consistency of our tag set. some inconsistencies were identified during annotation, these were addressed through iterative refinement and feedback from annotators, resulting in improved tagging accuracy and greater reliability of the tag set.

## 7 Challenges During POS Tagging of Pahari language

Building linguistic resources for under-resourced languages presents both challenges and opportunities. While South Asian languages have limited resources, Pahari lacked them entirely. In developing a POS-tagged corpus for Pahari, we encountered several key challenges in Table 18.

**Table 18:** Challenges in Pahari POS Tagging.

This consolidated table highlights the principal contextual ambiguities encountered during Pahari POS annotation. By aligning each sentence with its word-level tags and translations, it demonstrates how identical lexical forms assume different grammatical functions across contexts, underscoring the necessity of context-aware tagging for morphologically rich and low-resource languages.

## 8 Results & Discussion

The final distribution of tags across the Pahari POS-tagged corpus reflects the depth, complexity, and success of the annotation project. The corpus, consisting of approximately 200,000 tokens, was annotated using a carefully designed 33-tag system developed specifically for the grammatical and syntactic structures of Pahari. The final POS-annotated Pahari corpus contains 9205 sentences and 192,168 tokens, with a vocabulary size of 16,676 unique word types. Sentence lengths range from 3 words to 119 words, with an estimated average sentence length of approximately 21 words. The sentence length distribution is right-skewed, with most sentences occurring between 14 and 18 words, as represented in the Table 19. These statistics demonstrate the linguistic richness and structural diversity of the corpus, making it suitable for POS tagging, language modeling, and broader NLP tasks.

**Table 19:** Summary Statistics of the Pahari POS Corpus.

Statistic	Value
Total Sentences	9205
Total Tokens (Words)	192,168
Vocabulary Size (Unique Word Types)	16,676
Shortest Sentence Length	3 words
Longest Sentence Length	119 words
Estimated Average Sentence Length	∼21 words
Average Sentence Length (Most Frequent)	∼14–18 words

**Fig. 4:**
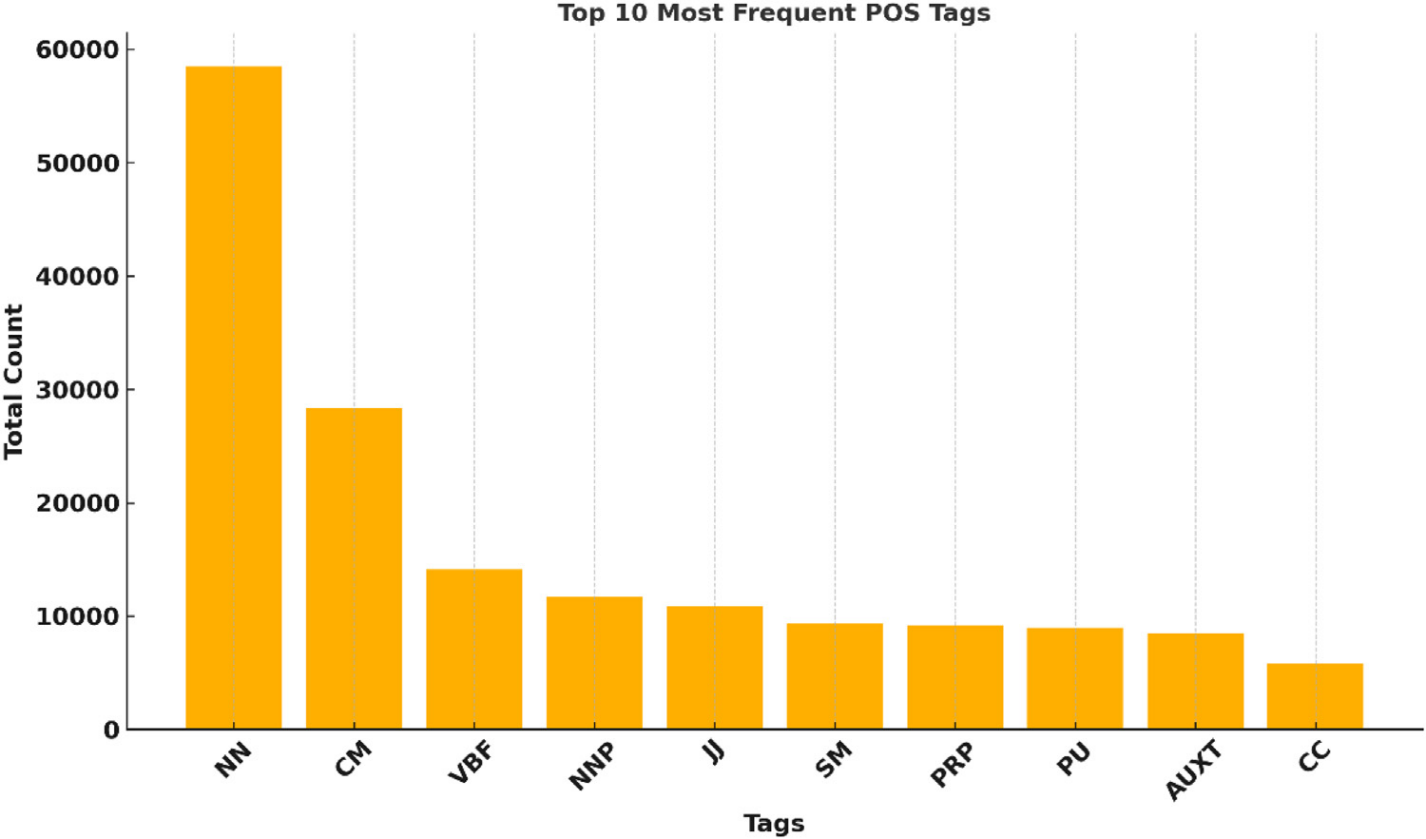
Top 10 tags.

The tag frequencies presented in the Fig. 4 revealed important insights into the linguistic profile of the language. As expected, common nouns (NN) dominate the corpus with 58,494 occurrences, highlighting the noun-heavy morphology characteristic of Indo-Aryan languages. Finite verbs (VBF) also appear frequently (14,122 instances, underscoring the dynamic action-oriented nature of Pahari sentences. Table 20 represents the final count of Part of Speech dataset.

**Table 20:** Final Tag count of Pahari POS Dataset.

S.No	TAGS	Total Count
1	ADV	1434
2	APNA	1227
3	AUXA	3297
4	AUXP	728
5	AUXT	8477
6	CC	5768
7	CD	3265
8	CM	28,321
9	CPR	145
10	FF	401
11	FR	44
12	I	105
13	JJ	10,855
14	NEG	749
15	NN	58,494
16	NNC	647
17	NNP	11,726
18	NNPC	397
19	OD	681
20	PRP	9194
21	PRR	1914
22	PRS	362
23	PRT	2897
24	PU	8965
25	Q	867
26	SC	2139
27	SCP	194
28	SM	9314
29	SYM	3812
30	VALA	634
31	VBF	14,122
32	VBI	4218
33	UNK	11

Auxiliary verbs were carefully separated into aspectual (AUXA), progressive (AUXP), and tense-based (AUXT) categories, with notable occurrences, particularly AUXT (8477 instances), reflecting the rich expression of tense and aspect in Pahari syntax. Proper nouns (NNP, NNPC) combined account for a substantial portion of the corpus, supporting the need for Named Entity Recognition (NER) systems tailored to multi-word entities common in South Asian languages. The high frequency of semantic markers (CM) (28,321 instances) also points to the crucial role of postpositions and relational particles in structuring meaning in Pahari discourse.

**Fig. 5:**
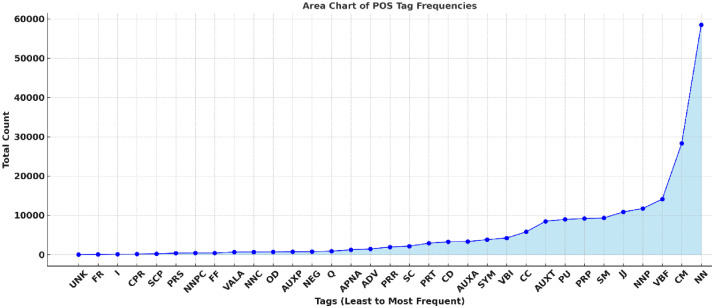
Area Chart of Whole Dataset.

Linguistic categories such as quantifiers (Q), ordinal numbers (OD), and fractional numbers (FR) were carefully annotated, revealing the nuanced expression of quantity and ranking in everyday Pahari communication. Special categories such as foreign fragments (FF) and symbols (SYM), though less frequently encountered, played a crucial role in preserving the integrity of modern borrowed terms and script variations within the corpus. Area chart of ll tags is presented in Fig. 5.

The annotation and development of a Part-of-Speech (POS) tagged corpus for the Pahari language uncovered several significant linguistic, computational, and methodological insights. Most notably, this study produced the first extensive POS-tagged dataset for Pahari, an under-resourced Indo-Aryan language spoken across Azad Jammu and Kashmir, Pakistan, and neighboring regions. The resulting corpus, comprising approximately 200,000 tokens, was meticulously annotated using a newly developed POS tagset tailored to reflect Pahari’s unique grammatical and syntactic structures.

To accommodate language-specific features, a 33-tag system was carefully constructed by adapting elements from Urdu, Hindi, and Punjabi POS tagsets, along with essential modifications unique to Pahari. Particular emphasis was placed on distinguishing finite verbs (VBF) from auxiliary verbs (AUXA, AUXP, AUXT) to accurately capture tense, aspect, and modality, and on identifying compound noun constructions (NNC, NNPC), which are highly prevalent in Pahari yet often underrepresented in other South Asian tagging systems.

The linguistic compatibility of the tagset with other South Asian languages ensures its potential for cross-linguistic research and the development of multilingual NLP tools.

Annotation reliability was confirmed through a high inter-annotator agreement score of 92.3 % (Cohen’s Kappa), underscoring both the clarity of annotation guidelines and the robustness of the tagset. A rule-based, iterative validation process was employed to resolve ambiguities, particularly in cases where words alternated roles between nouns, adjectives, finite verbs, and numerals. Rather than relying on superficial pattern-based tagging, annotators applied a context-sensitive approach, ensuring deeper grammatical precision appropriate for a morphologically rich language like Pahari.

This corpus and tagset establish a foundational computational resource for advancing NLP in Pahari. They enable a range of downstream tasks including morphosyntactic parsing, syntactic treebank construction, Named Entity Recognition (NER), cross-lingual language modeling, and machine learning applications such as POS tagging and speech-to-text systems.

Beyond technical advancements, this work serves an important sociolinguistic purpose. It contributes to the development of educational resources, supports language preservation and revitalization efforts, and promotes digital inclusion for the Pahari-speaking community thereby fostering their cultural and linguistic identity and addressing a long-standing gap in computational language resources.

## 9 Conclusion

The present study successfully developed the first large-scale Part-of-Speech (POS) tagged corpus for the Pahari language, addressing a significant gap in linguistic resources for this underrepresented language. A novel tagset comprising 33 categories was carefully designed to reflect the unique grammatical structure of Pahari. Approximately 200,000 tokens were manually annotated, achieving a high inter-annotator agreement of 92.3 %, which demonstrates the reliability and robustness of the annotation guidelines.

Beyond its technical contributions including applications in morphosyntactic parsing, Named Entity Recognition (NER), and cross-lingual Natural Language Processing this study plays a critical role in preserving the Pahari language by creating a structured and accessible linguistic resource. By resolving challenges such as contextual ambiguity in the use of verbs, nouns, adjectives, and auxiliary constructions, it establishes a solid foundation for future computational and linguistic research.

Overall, this work serves as both a computational tool and a cultural asset, helping ensure that the Pahari language maintains a visible and active presence in the evolving landscape of digital technologies. Furthermore, it offers a scalable model for developing linguistic resources for other low-resource languages globally.

## CRediT Author Statement

**Nadia Mushtaq Gardazi:** Data curation, Methodology, Visualization, Writing – Original draft preparation. **Dr. Kamran Malik:** Conceptualization, Methodology, Supervision. **Dr. Ali Daud:** Funding acquisition, Methodology, Supervision, Writing – Review & Editing.

## Declaration of Competing Interest

The authors declare that they have no known competing financial or personal interests that could have appeared to influence the work reported in this paper.

## Data Availability

Mendeley DataPahari Dataset (Original data). Mendeley DataPahari POS Dataset (Original data).
